# Frequency of Vertebral Fractures in Patients presenting with Hip Fractures

**DOI:** 10.12669/pjms.36.ICON-Suppl.1709

**Published:** 2020-01

**Authors:** Muhammad Amin Chinoy, Salman Javed

**Affiliations:** 1 Prof. Dr. Muhammad Amin Chinoy, FRCS. Department of Orthopedics, The Indus Hospital, Karachi, Pakistan; 2 Dr. Salman Javed, MBBS. Resident, Department of Orthopedics, The Indus Hospital, Karachi, Pakistan

**Keywords:** Hip fracture, Vertebral fractures, morbidity

## Abstract

**Objective:**

To determine the frequency of vertebral fractures in patients presenting with hip fractures.

**Methods:**

This prospective study was conducted at The Indus Hospital, Karachi, from May 2018 to November 2018. All patients above 40 years presenting with hip fractures were enrolled and a dorsal lumbar lateral view radiograph was obtained to investigate for vertebral fractures. Data was entered and analyzed using SPSS. Post-stratification, Chi-square/Fisher exact test was applied as appropriate to assess the significant association. P value of ≤0.05 was considered significant.

**Results:**

Three hundred thirty five patients were enrolled. Of these, 189 (56.4%) were females and 165 (49.3%) presented with neck of femur fractures. Out of 335 hip fractures patients, 77 (23%) were found to have concomitant vertebral fractures, with 73 (96.1%) having a compression fracture. T12 was the most common vertebra involved and 68.8% of patients were asymptomatic. Co-morbid conditions were statistically significantly associated with vertebral fractures.

**Conclusion:**

There is a high prevalence of asymptomatic vertebral fractures in our population, but low compared to studies from western countries. There is a need to evaluate these fractures separately for the prevention of morbidity and mortality.

## INTRODUCTION

Vertebral fractures (VFs) are often asymptomatic and remain undiagnosed until patients experience a subsequent more distressing and disabling fracture. Hip fractures are associated with increased morbidity and mortality.^1^ and cause not only considerable pain and disability but are a burden for the caregivers and community because of expensive rehabilitation, poor quality of life and associated high mortality. Nearly 1.6 million hip fractures are reported each year worldwide and are expected to rise at least three-fold by 2050.^2^,^3^

The Pakistan Society for the Rehabilitation of the Disabled (PSRD), a key foundation of orthopedic, medical and surgical health care and rehabilitation in the country, quotes that 50–75% cases of hip fractures are surgically cared for in urban areas.^3^ Low and middle income countries (LMICs) such as Pakistan are poorly equipped to handle this liability due to high costs of treatment and rehabilitation. Post-operative rehabilitation is often suboptimal and difficult due to the patient’s age and lack of commitment to prolonged therapy.

A pre-existing VF may affect rehabilitation. Vertebral compression fractures are typically caused by osteoporosis and vary from mild to severe.^4^ A range of clinical factors, including advanced age and sex, are independent risk factors that can predict the incidence of VF. Vertebral compression fractures of the thoracolumbar spine are common with advancing age, with prevalence approaching 40% by 80 years.^2^

Vertebral compression fractures are mostly asymptomatic. However if symptomatic, they can be the source of significant pain, affecting performance of daily activities and may cause mortality. Conservative treatment includes bed rest, pain control, and physical rehabilitation therapy. In patients who do not respond to initial treatment, interventional procedures such as vertebroplasty can be considered. Most VFs occur at the level of thoracic and lumber spine. Chitragar et al.^5^ reported that 62% of the VFs occur between T11 and L2 vertebrae. Patients who have had a VF once in a lifetime have 5 - 12.6x greater risk of having a further VF; and 2.3 - 3.4x higher risk of developing hip fractures.^6^ Worldwide, documentation of VFs preceding a second fracture is poor making its prevalence and incidence unknown.

Vertebral injuries, when accompanied with hip fractures, are not only associated with pain and prolonged recovery but also can cause other healthcare complications such as deep venous thrombosis due to extended periods of immobilization.^7^ They are often unnoticed and thus rehabilitation even after surgery is poor. Identifying these VFs at the right time is, therefore, of utmost importance to improve the care of such patients. To the best of our knowledge, no study has been conducted in our region demonstrating the presence of VFs in patients with hip fractures. Therefore, we set out to determine the frequency of vertebral fractures in patients presenting with hip fractures.

## METHODS

The study was prospectively conducted at The Indus Hospital, from May 2018 to November 2018, after obtaining ethical approval under IRD_IRB_2018_03_008. All patients aged 40 years and above presenting with hip fractures were enrolled in the study after obtaining informed consent. Those patients with a previous history of spinal or vertebral illness, injury or surgery were excluded. A proforma was filled to collect age, comorbid and medications patients were using. A lateral thoracic and lumbar spine radiograph was performed to assess VF and level of vertebral fracture. Sample size was calculated using open epi software with a 95% confidence interval and 5% desired precision, assuming 68% prevalence of VF in elderly patients with hip fracture^8^ and was found to be 335. Data were entered and analyzed using SPSS version 24.0. Mean ± SD was computed as appropriate for age. Frequency and percentages were computed for gender, type of fractures and comorbidities. Independent sample T-test/Mann-Whitney U test was applied as appropriate to assess the significant difference in age between the patients who had vertebral fractures and who did not have vertebral fractures. ANOVA/Kruskal Wallis test was applied as appropriate to assess a significant difference in age between the types of fracture. Effect modifier/confounder was assessed through stratification. Post-stratification, Chi-square/Fisher exact test was applied as appropriate to assess the significant association. P-value ≤0.05 was considered significant.

## RESULTS

A total of 335 patients were enrolled in the study. Of these, 146 (43.6%) were males and 77 (23%) were found to have vertebral fractures (Table-I), with 73 (96.1%) having compression fracture and 5 (6.6%) translation fracture. Of these 77 patients, 25 (32.5%) had T12 vertebral fracture, 20 (26%) had L1, 17 (22.1%) had L5 vertebral fracture. The majority (n=53, 68.8%) of the patients with vertebral fractures had no symptoms while 24 (31.2%) had back pain (Figure-1). The median (IQR) age of the patients was 65 years (55-73) (Table-II) with a significant difference in the median age between both the genders and patients with and without vertebral fracture. Women with hip fractures were older than men (Median: 66 vs 62.5, p=0.009,). Patients who had concomitant vertebral fractures were younger than those who did not (Median age: 62 vs 72, p=0.000.

**Table I:** Descriptive statistics of the enrolled patients.

***Age in years***	
Mean ± SD	63.6 ± 12.7
Min-Max	40 – 85
Median (IQR)	65(55 - 73)
***Gender; n (%)***	
Male	146 (43.6)
Female	189 (56.4)
***Type of hip fracture; n (%)***	
Intertrochanteric fracture	142 (42.4)
Neck of femur fracture	165 (49.3)
Sub trochanteric femur fracture	28 (8.4)
***Vertebral fracture; n (%)***	
No	258 (77)
Yes	77 (23)
***Comorbid; n (%)***	
Cancer	6 (1.8)
CVA	16 (4.8)
Diabetes	55 (16.4)
Hepatitis C	6 (1.8)
HTN	79 (23.6)
IHD	11 (3.3)
TB	3 (0.9)
CKD	4 (1.2)
Other	11 (3.3)
None	203 (60.6)
***Medications; n (%)***	
Antihypertensive	72 (21.5)
Aspirin	12 (3.6)
Insulin	6 (1.8)
Oral Hypoglycemic	44 (13.1)
Steroids	3 (0.9)
Other	16 (4.8)
***Type of vertebral fractures; n (%)***	
Compression fracture	73 (96.1)
Translation fracture	4 (6.6)

**Table II:** Differences in age between genders, type of hip fractures and vertebral fracture.

	Mean ± SD	Min-Max	Median (IQR)	P-value
***Gender***				
Male	61.4 ± 13	40 - 85	62.5(50 - 71)	0.009*^ɬ^
Female	65.3 ± 12.2	40 - 85	66(56 - 75)
***Type of hip fractures***				
Intertrochanteric fracture	67.5 ± 12.4	40 - 85	70(60 - 79)	0.000**^ǂ^
Neck of femur fracture	61.6 ± 12	40 - 85	61(52.5 - 70)	
Sub trochanteric femur fracture	55.4 ± 11.5	40 - 74	55(45 - 66.5)
***Vertebral fractures***				
Yes	61.5 ± 12.5	40 - 85	62(50 - 70)	0.000**^ɬ^
No	70.5 ± 10.9	40 - 85	72(62 - 80)	

* P-value<0.05,

** P-value<0.0001,

ɬ Mann-whitney U test,

ǂ Kruskal Wallis test.

Nearly half (n=165; 49.3%) of patients presented with neck of femur fractures, followed by intertrochanteric fractures (n=142; 42.4%) and 28 (8.4%) subtrochanteric femur fractures (Table-I). It was found that patients who presented with intertrochanteric fracture were older and had median age 70 years (IQR: 55 – 73), while those presenting with the neck of femur and subtrochanteric femur fractures had a median age of 61 and 55 years respectively (p=0.000, Table-II). There was no significant difference in the distribution of vertebral fractures between both the genders (20.5% vs 24.9%, p=0.351. However, a significantly higher proportion of vertebral fractures were observed in intertrochanteric hip fractures patients (32.4%), followed by patients with neck of femur fracture (17%) and subtrochanteric femur fractures (10.7%) (p=0.002. In addition, more vertebral fractures were observed in patients of age over 65 years as compared to those less than 65 years (31.6% vs 13.7%, p=0.000. Moreover, a higher proportion of vertebral fractures were found in patients who had chronic kidney disease, followed by hepatitis C, cancer and cerebrovascular accident (75%, 66.7%, 33.3%, and 31.3% respectively, p=0.049. No significant association was observed between medication and vertebral fractures (p=0.217, Table-III).

**Table III:** Distribution of vertebral fracture according to gender, type of hip fractures, age groups and comorbid.

	Vertebral fractures			

	No	Yes	Total	p-value

	N (%)	N (%)	N (%)	
***Gender***				
Male	116 (79.5)	30 (20.5)	146 (100)	0.351
Female	142 (75.1)	47 (24.9)	189 (100)	
Total	258 (77)	77 (23)	335 (100)
***Type of hip fractures***				
Intertrochanteric fracture	96 (67.6)	46 (32.4)	142 (100)	0.002*
Neck of femur fracture	137 (83)	28 (17)	165 (100)	
Sub trochanteric femur fracture	25 (89.3)	3 (10.7)	28 (100)	
Total	258 (77)	77 (23)	335 (100)
***Age in years***				
< 65 years	139 (86.3)	22 (13.7)	161 (100)	0.000**
> 65 years	119 (68.4)	55 (31.6)	174 (100)	
Total	258 (77)	77 (23)	335 (100)
***Comorbid***				
Cancer	4 (66.7)	2 (33.3)	6 (100)	0.049*
CVA	11 (68.8)	5 (31.3)	16 (100)	
Diabetes	44 (80)	11 (20)	55 (100)	
Hepatitis C	2 (33.3)	4 (66.7)	6 (100)	
HTN	64 (81)	15 (19)	79 (100)	
IHD	9 (81.8)	2 (18.2)	11 (100)	
Other	9 (81.8)	2 (18.2)	11 (100)	
TB	2 (66.7)	1 (33.3)	3 (100)	
CKD	1 (25)	3 (75)	4 (100)
***Medications***				
Antihypertensive	58 (80.6)	14 (19.4)	72 (100)	0.217
Aspirin	9 (75)	3 (25)	12 (100)	
Insulin	3 (50)	3 (50)	6 (100)	
Oral Hypoglycemic	38 (86.4)	6 (13.6)	44 (100)	
Other	12 (75)	4 (25)	16 (100)	
Steroids	1 (33.3)	2 (66.7)	3 (100)	

* P-value<0.05,

** P-value<0.0001, Chi-square test

## DISCUSSION

In this study, 23% of patients presenting for hip fractures also had a concomitant VF. Our findings were comparable to that of Sadat-Ali et al who reported a VF in 24.7% of the patients in Saudi Arabia, with more prevalence being in females.^9^ As stated by a Norwegian study in 2012, the prevalence of vertebral fractures varies according to age. The study reported the prevalence of VF in women as 3.4% when age <60 years, 11.1% with age 60-69 years and 19.2% with ≥70 years; whereas the prevalence was higher for men; 7.6% with age <60 years, 11.9% with age 60-69 years and 20.3% with ≥70 years. The average prevalence in men and women was reported to be 13.8% and 11.8% respectively.^10^ Our results with respect to age are consistent with this, as frequency in patients aged over 65 was greater than those aged less this (31.6% vs 13.7%). The similarity is likely due to the presence of osteoporosis in patients of both studies. However, our study didn’t show gender difference in VF as seen in other study. A study conducted in Spain (2007) assessed elderly women for VF who were admitted to the hospital due to hip fractures. Here, the prevalence of VFs in elderly women with hip fractures was 62.6%.^11^ An Italian study (2016) reported the prevalence of VF along with hip fracture as 60% in elderly males and 69% in females, with overall prevalence of 68%.^8^ This difference in prevalence of vertebral fractures could be due to the different genetic background of the population group compared with our population and also due to geographical location.

In our study, patients with vertebral fractures were largely (68%) asymptomatic. Chitragar et al.^5^ conducted a study in Saudi Arabia, reporting one-fourth of patients presenting with hip fractures had concomitant asymptomatic vertebral fractures. Gallacher et al.^12^ also found that 25% of patients presenting with non-vertebral fracture also had a simultaneous, asymptomatic VF.

We found T-12 vertebra to be the most commonly affected vertebra (32.2%). Similar findings were reported previously, with the prevalence of vertebral compression fractures being more common in the mid-thoracic region and thoracolumbar transition.^9^,^13^

The presence of vertebral fractures along with hip fractures are often due to osteoporosis and poor bone quality. Thus not only it is important to know the prevalence of osteoporotic fractures for better rehabilitation but is also significant when fracture fixation is planned. The implant selection for the management of osteoporotic proximal femur fractures should include evaluation of the fracture pattern, pre-existing bone quality, mobility, presence of arthritis and mental status as well as surgeon experience. In addition, there should be consideration of implant cost and outcomes of specific implants.^14^ Co-morbid conditions of the patients were also noteworthy in our study, with hypertension being the most common.

Rehabilitation is also a major concern in patients having asymptomatic vertebral fractures. These fractures in combination can affect the quality of life and affect morbidity and mortality. It is well-known that vertebral fractures are risk factors for incident hip fracture and predictors of future risk of vertebral and hip fractures.^15^ Thus it is of paramount importance to consider other asymptomatic skeletal problems of a patient presenting with hip fracture, such as vertebral fractures, lumber lordosis, thoracic kyphosis, in order to not only decrease the mortality but also enable better rehabilitation and reduce further social, economic and emotional stress on the family and by hence on the community.

## CONCLUSION

The frequency of asymptomatic vertebral fractures in our population, is comparable to that of other studies from South Asia but not when compared to data from the Western world. This may be either due to environmental factors affecting the geographical location or different genetic background between South Asian population and Caucasians, since all other factors such as mode of investigation were similar. Vertebral fractures were significantly associated with age and co-morbid condition of the patient.

### Authors’ Contribution

**MAC:** Developed the study design, interpretation of data, drafting the manuscript and revising it.

**SJ:** Contributed to study design, helped in data collection, drafting the manuscript and revising it.
